# The Efficacy of Electron Beam Irradiated Bacterial Cellulose Membranes as Compared with Collagen Membranes on Guided Bone Regeneration in Peri-Implant Bone Defects

**DOI:** 10.3390/ma10091018

**Published:** 2017-09-01

**Authors:** So-Hyoun Lee, Sung-Jun An, Youn-Mook Lim, Jung-Bo Huh

**Affiliations:** 1 Department of Prosthodontics, Dental Research Institute, Institute of Translational Dental Sciences, BK21 PLUS Project, School of Dentistry, Pusan National University, Yangsan 50612, Korea; romilove7@hanmail.net (S.-H.L.); 2 Advanced Radiation Technology Institute, Korea Atomic Energy Research Institute, 1266 Sinjeong-dong, Jeongeup-si, Jeollabuk-do 56212, Korea; asj@kaeri.re.kr (S.-J.A.); ymlim71@kaeri.re.kr (Y.-M.L.)

**Keywords:** guided bone regeneration (GBR), bacterial cellulose membrane (BCM), electron beam irradiation (EI), resorbable barrier membrane, animal study

## Abstract

Bacterial cellulose (BC) is a natural polysaccharide produced by some bacteria, and consists of a linear polymer linked by β-(1,4) glycosidic bonds. BC has been developed as a material for tissue regeneration purposes. This study was conducted to evaluate the efficacy of resorbable electron beam irradiated BC membranes (EI-BCMs) for guided bone regeneration (GBR). The electron beam irradiation (EI) was introduced to control the biodegradability of BC for dental applications. EI-BCMs had higher porosity than collagen membranes (CMs), and had similar wet tensile strengths to CMs. NIH3T3 cell adhesion and proliferation on EI-BCMs were not significantly different from those on CMs (*p* > 0.05). Micro-computed tomography (μCT) and histometric analysis in peri-implant dehiscence defects of beagle dogs showed that EI-BCMs were non-significantly different from CMs in terms of new bone area (NBA; %), remaining bone substitute volume (RBA; %) and bone-to-implant contact (BIC; %) (*p* > 0.05). These results suggest resorbable EI-BCMs can be used as an alternative biomaterial for bone tissue regeneration.

## 1. Introduction

Natural biopolymers have been at the focus of research on biocompatible materials and devices because of their biomimetic properties; examples of such materials include collagen, hyaluronic acid, gelatin, and cellulose provided [1]. Cellulose is the most plentiful and widespread biopolymer on earth and structurally resembles natural extracellular matrix (ECM) [2]. It is a natural polysaccharide produced by plants and some bacteria and consists of a linear polymer joined by β-(1,4) glycosidic linkages [3,4].

**Figure 1:**
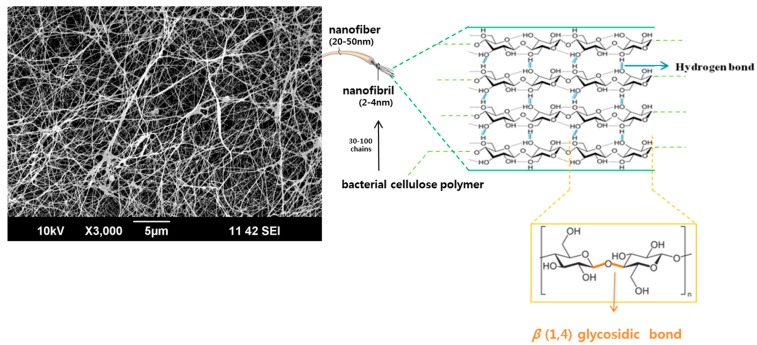
Schematic of bacterial cellulose structure.

BC was discovered as a gelatinous membrane on culture medium surfaces during *Mycoderma aceti* fermentation by Brown in 1886 [5,6]. Bacterial celluloses (BCs) are composed of ultrafine three-dimensional (3D) networks of ribbon-shaped cellulose nanofibers composed of nanofibrils of 2–4 nm in diameter, and are structurally similar to plant celluloses (PCs) [7,8] (Figure 1). However, BCs have better physicochemical properties than PCs [4], such as higher wet strengths [3], interconnected 3D porous surfaces [9], crystallinities [10], and water holding capacities [11]. Furthermore, BC fibers, unlike PC fibers, do not need chemical treatments because they are free of hemicellulose, lignin and pectin [12]. In addition, BCs have excellent biocompatibilities [13]. For these reasons, BCs are recognized as being suitable for the tissue regeneration of body organs and have been developed for biomedical applications, such as wound dressings and artificial skin, drug release systems, blood vessel and nerve reconstruction, stent coverings, and for promoting bone tissue regeneration [14].

BCs have attracted the attention of researchers in the dental field because of their 3D porous structures, excellent tensile strengths, and biocompatibilities [15]. Research has been conducted from the 1980s [16,17] on the application of BCs for bone tissue regeneration; examples of research topics include, BC hydroxyapatite nanocomposites [18], incorporation of growth factor in BC scaffolds [19], and the use of BCs as barrier materials [20]. Guided bone regeneration (GBR) is widely used to investigate dental implant support in bone defects [21]. The barrier membranes used for GBR should have the characteristics of cell occlusiveness (to prevent epithelial tissue down-growth inside bone defects), space making (to allow new bone formation), wound stabilization, support for stable bone regeneration, and promote bone tissue regeneration [22,23].

Barrier membranes are classified as resorbable and non-resorbable according to their biodegradabilities in vivo [24]. Non-resorbable barrier membranes, such as those made of expanded polytetrafluoroethylene (e-PTFE) and titanium mesh, possess excellent mechanical stiffness and space-maintenance ability, but require secondary surgery procedure to remove them and complications like premature membrane exposure [25,26]. Resorbable barrier membranes comprised of inorganic ceramics, polylactic acid, polyglycolic acid or collagen have been introduced to overcome the limitations of non-resorbable barrier membranes [27]. Collagen membranes (CMs) are most popular in the dental field because they do not cause tissue damage and improve soft tissue healing [28]. The 3D porous structure of BCs is similar to that of CMs [15]. While the protein components of CMs are likely to beimmunogenic, the neutral polysaccharides that compose BCs are not [29]. In particular, BC is a cost-effective biopolymer, and is expected to provide a novel alternative to CMs as a barrier material for GBR [18]; thus, its mechanical and biological properties have been examined to determine its suitability as for resorbable barrier membranes.

However, BC is near non-degradable in the human body due to its high degree of crystallinity and absence of enzymes that break the β-(1,4) glycosidic linkage of cellulose [30,31]. Some in vivo studies have reported that BC membranes implanted in the subcutaneous tissue of rats were not resorbed after 12 weeks, and induced mild inflammatory response until 30 days after transplantation with no signs of foreign bodies around implanted membranes [32,33]. In another study, BC membranes implanted in the dura mater of dogs partially disappeared at 270 days post-transplantation [34], and BC grafted in the nasal dorsum of rabbits exhibited slight fragmentation at six months without foreign body reaction [35]. Although non-degradable BC is an effective scaffold for long term support, its lack of biodegradability limits its use as a resorbable barrier membrane [36]. Several suggestions have been made to improve the biodegradability of BC. In vitro, the formation of fuzzy aggregates and BC fiber fragmentation were identified after immersion for 8–12 weeks in phosphate-buffered saline (PBS) at pH 7.25 and 37 °C [37,38]. An in vivo study [39] on BC-hydroxyapatite (HA) nanocomposite membranes in rat tibia showed the sizes of HA particles and BC nanofibers determined resorption. Li et al. [40] reported that amorphous regions of BC chemically modified by periodate oxidation were converted to biodegradable 2,3-dialdehydebacterial cellulose (DABC). Czaja et al. [41,42] noted that BC membranes pre-irradiated with γ-radiation and by periodate oxidation degraded most rapidly during the first 2–4 weeks. Hu and Catchmark [43] suggested that biodegradability could be enhanced by incorporating cellulose-degrading enzymes into the nanostructure of BC. However, these methods are limited by difficulties of controlling biodegradation and potential cytotoxicity due to the presence of residual chemicals in BC [44].

In our previous study, untreated BCMs was founded to have higher mechanical strength than CMs but to have several limitations such as low cell response and poor biodegradability for application as a resorbable barrier membrane of GBR [45]. Therefore, in the present study, an electron beam irradiation (EI) process was used to control BC-cell interactions and biodegradability of BC. Irradiation processes based on the use of gamma rays, ion beams, or electron beams are widely used to regulate the thermal, mechanical, and chemical properties of biopolymers [46]. High energy electron beams have been used to cross-link or degrade polymers, and kill microorganisms [47] since this technique effectively breaks polymer chains [48]. The greatest advantage of EI is that it does not require chemicals [49]. Decomposition can also reduce polymer molecular weight, reduce mechanical properties, and increase solubility, and, thus, careful processing is required when using EI to treat resorbable BC membranes [50,51]. However, few studies have investigated the mechanical and biological properties of electron beam irradiated BC membranes (EI-BCM).

Therefore, the purpose of this study was to evaluate the efficacy of EI-BCMs as potential resorbable barrier membrane for GBR by comparing with CMs. The mechanical and biological properties of EI-BCMs were investigated with SEM, wet tensile strength, porosity and in vitro cell study. Moreover, bone regeneration effect of EI-BCMs was evaluated through micro-computed tomography (μCT) and histometric analysis in a peri-implant dehiscence bone defect model of large animal.

## 2. Results

### 2.1. The Results of the Mechanical Studies

#### 2.1.1. Scanning Electron Microscope (SEM) Morphologic Analysis

**Figure 2:**
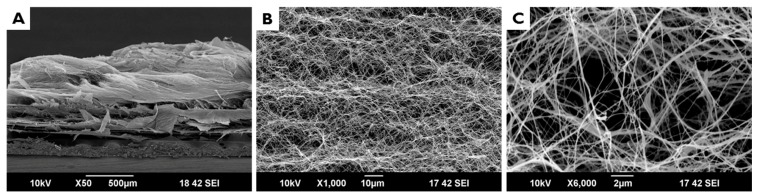
Cross sectional SEM images of non-pressed, electron beam irradiated BC membrane (EI-BCM) (Original magnification: 50× (**A**); 1000× (**B**); and 6000× (**C**)). EI-BCMs had a porous structure comprised of entangled nanofibers.

**Figure 3:**
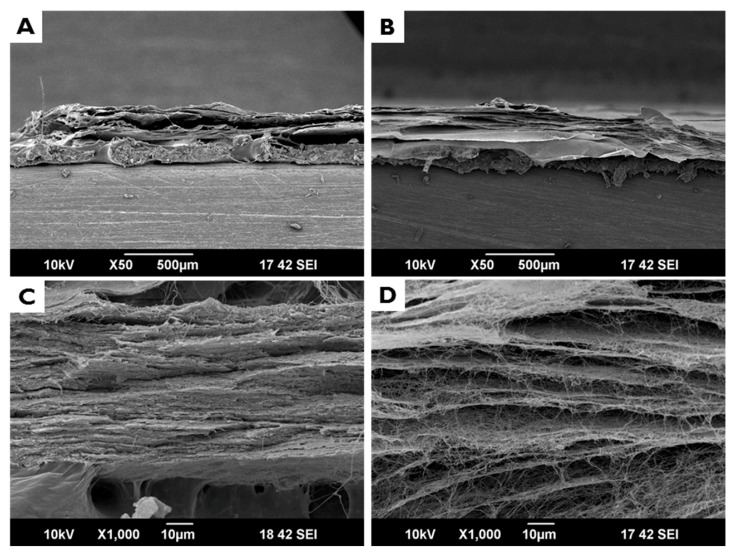
Cross sectional SEM images: (**A**,**C**) collagen membrane (CM); and (**B**,**D**) electron beam irradiated BC membrane (EI-BCM). EI-BCMs had a 3D structure cross-linked by nanofibers between each layers similar to the CM.

Figure 2 shows cross sectional SEM images of EI-BCM. The membrane had a porous structure comprised of entangled nanofibers. It was confirmed that EI-BCMs were compressed to a thickness similar to CMs and had a similar a multilayered lamellar structure (Figure 3). EI-BCMs had a 3D structure cross-linked by nanofibers between each layers similar to the collagen membrane (CM).

#### 2.1.2. Measurement of Mechanical Strength

**Figure 4:**
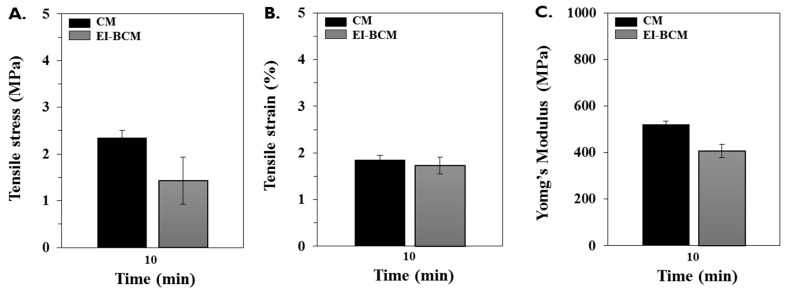
Mechanical properties of the CMs and EI-BCMs after a 10 min soak in water. No significant difference was found between the two membrane types: (**A**) tensile stress (MPa); (**B**) tensile strain (%); and (**C**) Young’s modulus (MPa). The mechanical properties of EI-BCMs were non-significantly different from those of CMs (*p* > 0.05).

The mechanical properties of CMs and EI-BCMs, including their wet tensile stresses, wet tensile strains, and Young’s moduli, are illustrated in Figure 4. Mean wet tensile stress of CMs and EI-BCMs were 2.42 ± 0.15 MPa and 1.43 ± 0.5 MPa, respectively; mean wet tensile strains were 1.90 ± 0.13% and 1.73 ± 0.18%, respectively; and mean Young’s moduli were 528.36 ± 19.21 MPa and 406.2 ± 28.57 MPa, respectively. The mechanical properties of EI-BCMs were non-significantly different from those of CMs (*p* > 0.05).

#### 2.1.3. Porosity Analysis

Mean porosities of CMs and EI-BCMs were 32.74 ± 0.05% and 92.421 ± 0.02%, respectively; mean pore diameters were 18 ± 0.15 µm and 28.05 ± 0.13 µm, respectively; and mean total pore areas were 0.015 ± 0.003 m^2^ and 0.091 ± 0.001 m^2^, respectively. These results confirmed EI-BCMs were more porous than CM.

### 2.2. Results of In Vitro

#### 2.2.1. Cell Viability

**Figure 5:**
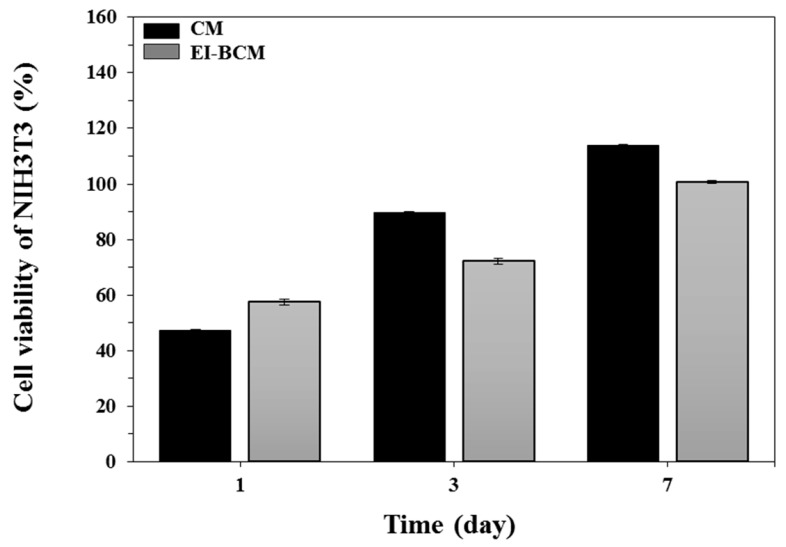
Viabilities of NIH3T3 cells on CMs and EI-BCMs as determined by CCK-8 assay at one, three and seven days. Viabilities of NIH3T3 cells on EI-BCMs were non-significantly different from those of CMs at each time point (*p* > 0.05).

CCK-8 assays were carried out on the effects of CMs and EI-BCMs on NIH3T3 cells to test cell proliferation and adhesion. As shown in Figure 5, number of NIH3T3 cells on CMs and EI-BCMs increased over seven days of incubation, and viabilities of NIH3T3 cells on EI-BCMs and CMs were non-significantly different at each time point.

#### 2.2.2. Immunofluorescent Staining Analysis of Cell Adhesion and Proliferation on the Membranes

**Figure 6:**
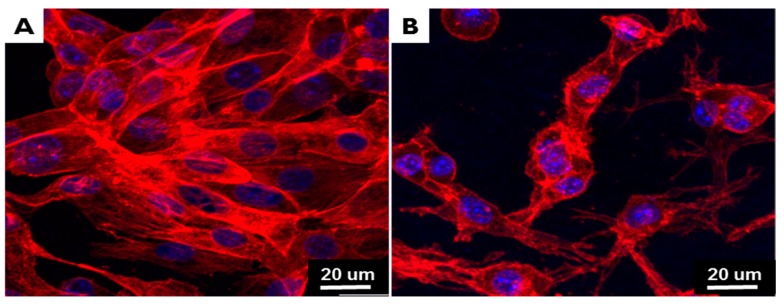
Immunofluorescent staining images of adherent NIH3T3 cells obtained by confocal microscopy: (**A**) CM; and (**B**) EI-BCM. NIH3T3 cell on EI-BCMs (**B**) had a long and straight F-actin similar to the CM.

The morphologies of cells on the membranes after seven day are represented in the immunofluorescence analysis (Figure 6). The abilities of membranes to provide NIH3T3 cell adhesion were evaluated by F-actin. Cells spread on membranes and actin fibers were long and straight. The cells integrated well with membrane nanofibers and cell growth was guided by the nanofiber structures of CMs and EI-BCMs.

### 2.3. The Results of In Vivo Studies

#### 2.3.1. Clinical Findings

Both experimental animals survived surgical procedures, and all eight implant sites in tow animal healed without evidence of inflammatory reactions. No membrane exposure or implant failure occurred during the eight-week healing period.

#### 2.3.2. Volumetric Analysis Using Micro-Computed Tomography (μCT)

**Figure 7:**
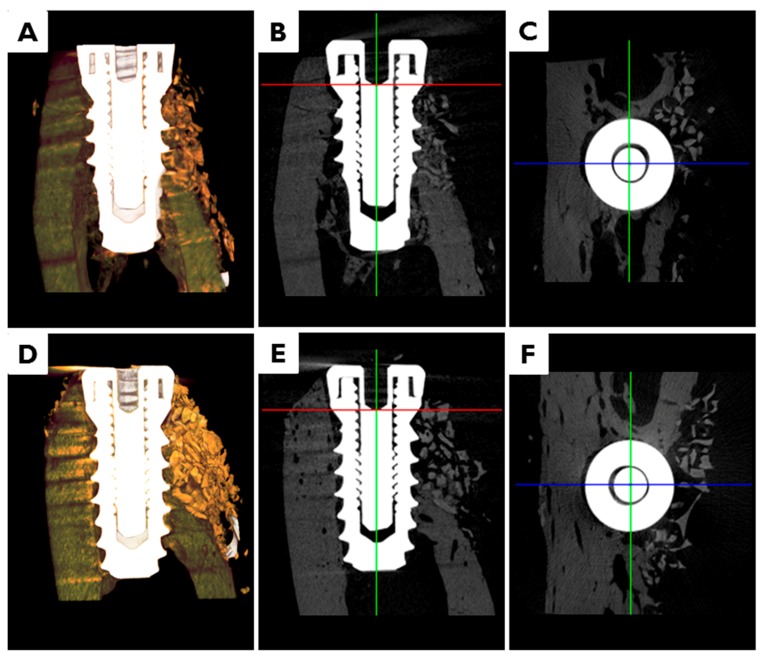
Micro-computed tomography (*μ*CT) images. In animals transplanted with: CM (**A**–**C**); or EI-BCM (**D**–**F**), bone graft materials were observed in peri-implant dehiscence defect areas at eight weeks after surgery. (**A**,**D**) 3D images; (**B**,**E**) 3D reconstructed images of mesiodistal sections; and (**C**,**F**) 3D reconstructed images of occlusal sections.

In animals transplanted with CM or EI-BCM, bone graft materials were observed in peri-implant dehiscence defect areas at eight weeks after surgery (Figure 7). Volumetric measurements are summarized in Table 1. Volumes of new bone (NBV, mm^3^), remaining bone substitute volumes (RBV, mm^3^), totally augmented volumes (TAV, mm^3^) and non-mineralized tissue volumes (NMV, mm^3^) were not significantly different in CM and EI-BCM transplanted animals at eight weeks (*p* > 0.05).

#### 2.3.3. Histologic Findings

**Figure 8:**
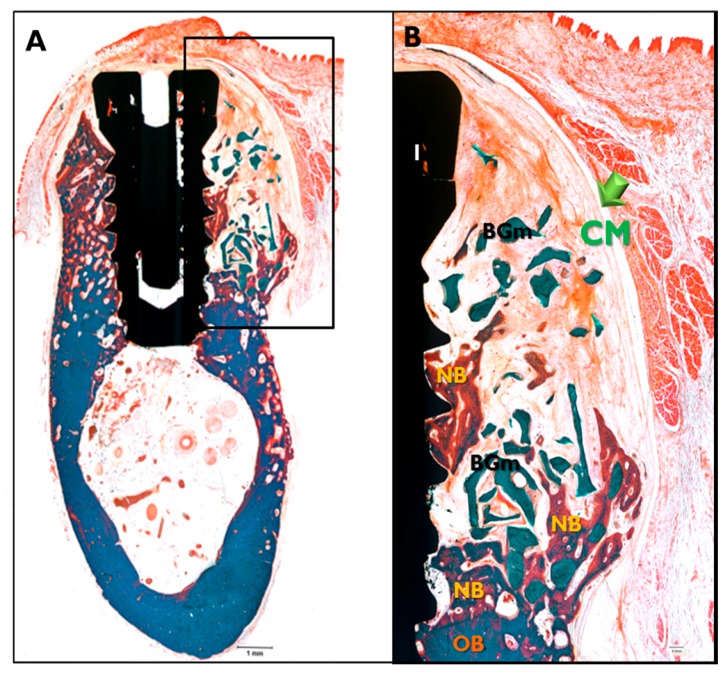
Histological sections specimen in CM transplanted animals. NB, new bone; BGm, bone graft material; OB, old bone; I, implant (Goldner Trichrome stained; original magnifications: 12.5× (**A**); and 40× (**B**)).

**Figure 9:**
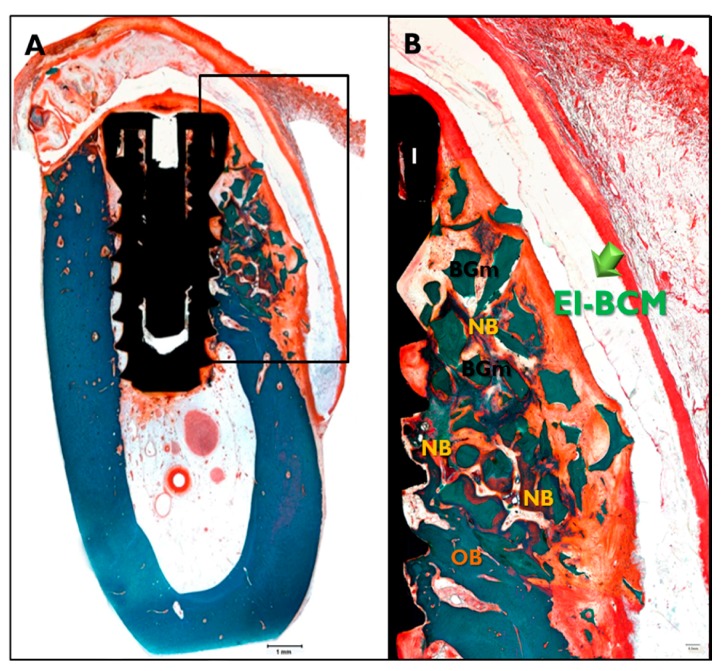
Histological sections specimen in EI-BCM transplanted animal. NB, new bone; BGm, bone graft material; OB, old bone; I, implant (Goldner Trichrome stained; original magnifications: 12.5× (**A**); and 40× (**B**)).

In the CM transplanted animals (Figure 8), a new bone formation was observed and fibrous connective tissues and graft materials were also observed in buccal peri-implant dehiscence defect areas. In some specimens, small amounts of membranes were observed. In the EI-BCM and CM transplanted animals (Figure 9), new bone, fibrous connective tissue, and graft materials were observed in the peri-implant dehiscence defect area. The EI-BC membranes remained at eight weeks in a similar pattern to the CM group, but expansion of the membrane was observed in some specimens.

#### 2.3.4. Histometric Analysis

Histometric results are summarized in Table 2. No significant difference was observed between CM and EI-BCM transplanted animals at eight weeks in terms of new bone areas (NBA; %), remaining bone substitute areas (RBA; %), or bone-to-implant contact (BIC; %) (*p* > 0.05).

## 3. Discussion

Bacterial cellulose (BC) is a transplantable biomedical material due to its superior crystallinity and purity, unique 3D porous nanofibrous network structure, structural stability, and properties during sterilization [1]. Davis et al. [52] suggested that the properties required for biopolymers for biomedical applications include: biocompatibility, cellular interaction, tissue development, biodegradability/bioabsorbability, interconnected porous structure, excellent mechanical strength and high wear resistance. BC has been investigated in these respects, but problems related to lack of biodegradation have yet to been solved. Therefore, in the present study, electron beam irradiation (EI) was investigated as a means of controlling BC biodegradation and the mechanical properties and biological effects of EI-BCMs were investigated.

BC is produced extracellularly by microorganisms, such as in the genera Gluconacetobacter, Azotobacter, Rhizobium, Salmonella, Escherichia, Pseudomonas, Alcaligenes and Sarcina [6]. Among these, G. xylium, G. hansenii, and G. pasteurianus, which are aerobic, rod shaped, Gram-negative bacteria, are known to be effective cellulose producers [7]. The present study was conducted using Gluconacetobacter hansenii TL-2C.

In the dental field, CMs are preferred resorbable barrier membranes that offer the advantages of biocompatibility, manageability, and in vivo resorbability [28]. In particular, the 3D structure of CM is similar to that of BC [12,13,14,15]. According to mechanical analysis, the EI process does not change the thickness or nanoporous network structure of BCMs [53], but the high energy electron beams cleave D-glucose chains resulting in cleavage of nanofibers [54]. The porosity of BC can be altered by metabolic source and culture conditions [1]. Furthermore, freeze-dried BCMs have been reported to be more porous and have more uniform pore sizes than hot air-dried BCMs [55]. In the present study, the lyophilization method [56] was used and the EI-BCMs produced had a mean porosity of 92% and a mean pore diameter of 28 µm, which is higher than that of CM (32% and 18 µm). In a previous study, we found unirradiated BCMs (59%) was more porous than CMs (34%) [45]. Natural biopolymers with porous structure, such as collagen and cellulose, are well known for their excellent biological functions [17]. Zellin and Linde [57] and Lundgren et al. [58] reported that barrier membranes with a pore size >25 µm provide better bone formation during the initial healing period than non-porous or smaller pore sized size membranes. Zaborowska et al. [32] suggested barrier membranes with nano-sized pores are more effective for cell attachment and differentiation, and that micro-sized pores enhance angiogenesis, transport of nutrients, cell migration, number of cell clusters within the pores, and increased the densities of mineral deposits.

In the in vitro cell studies, NIH3T3 fibroblasts, which are recommended as reference cell lines for the cytotoxicity testing of biopolymers, have been used to evaluate the cytocompatibility of two type of membranes [45]. Our cell viability results after seven days indicated that cell adhesion and proliferation on EI-BCMs was not significantly different from that on CMs (*p* > 0.05), whereas in a previous study, we observed cell viabilities on unirradiated BCMs were significantly lower than on CMs (*p* < 0.05) [45]. As shown by immunofluorescence analysis, the adherent cells on EI-BCM or CM were observed noticeable F-actin stress fiber, unlike polygonal form of cells on unirradiated BCM [17,32,59]. Cell responses to pure BC were reported to be weaker than those to CM due to the absence of charged groups in its polysaccharides [60]. The hydrophobic surface of pure BC makes it less immunogenic and enhances biocompatibility, but adversely affects cell tissue reactions due to lack of cell cognition [32]. The interfacial characteristics of biopolymers are important for cell attachment/adhesion, and several studies have been conducted to modify BC surfaces, using plasma [61] or irradiation [62], adhesive small signaling peptides [63] or amino acids, such as Arg-Gly-Asp (RGD), to enhance cell-BC interactions [64]. These surface modifications can change the mechanical and chemical properties of BC and affect wettability, porosity, and surface charges High energy irradiated BC membranes have been reported to enhance biological properties [46,54], and the improved cell responses shown by EI-BCMs are probably due to increased porosity and surface hydrophilicity [46,60]. 

Although EI has been shown to have important many advantages, it also reduces the mechanical properties of BC. The 3D network of BC is formed by hydrogen bonds between the cellulose chains of 20–50 nm nanofibers and a β-(1,4) glycosidic bonds between d-glucose [65]. These interactions account for the excellent tensile strength and water retention of BC, but also caused poor degradation, low solubility, and high crystallinity [13,65]. Buser et al. [66] mentioned that the mechanical strengths of barrier membranes is important for successful GBR. Furthermore, the resorbable membranes used for GBR should have sufficient mechanical strength to attach firmly to bone defects and prevent rupture during surgery [65,66,67,68]. In the present study, although polymer chain cleavage by EI reduced wet tensile strength, strain and Young’s modulus of BCM, these mechanical properties were low those of unirradiated BCM in our previous study [45] but similar to those of CM.

The results of μCT and histometric analysis in the large animal study showed bone regeneration and no significant difference between BCMs and CMs in terms of bone regeneration during the eight-week healing period. During the initial healing period, mild signs of inflammation, such as exudate and edema, were observed around grafted EI-BCMs in some animals, but no evidence of foreign body or microscopic inflammation response was observed at any time. These tissue responses BC materials have been reported in previous studies [13,33,34]. Interestingly, BC-associated infection rate in man is low, and as a result BC has been for wound and burn dressing [12,41]. In the present study, grafted EI-BCM integrated well with surrounding tissues, stabilized defects, and maintained adequate space for bone regeneration. However, we admit the eight-week healing period allowed was insufficient to confirm the complete biodegradation of EI-BCMs.

The histological findings obtained showed EI-BCMs had a tendency to expand slightly during the initial healing period, which we presume was because EI caused the cleavage of BC nanofibers, increased hydroxyl groups, surface hydrophilicity, and increase porosity [47,48,49,50]. In order to control this tendency, further studies are required on more, over a longer period of time, at an optimal radiation dose, on membranes of optimal thickness and porosity. 

This study was conducted to evaluate the efficacy of EI-BCM as a resorbable barrier membrane for GBR. According to the results, the EI process positively influenced porosity, cell adhesion/proliferation and biodegradation without altering the thickness or nanoporous network structure of BCM. Moreover, EI-BCM showed similar bone regeneration effect to collagen membrane in peri-implant dehiscence defects of beagle dogs (*p* > 0.05). Consequently, irradiated BC membrane by electron beam has the potential to replace existing resorbable barrier membrane such as collagen membrane.

## 4. Materials and Methods

### 4.1. Preparation of Barrier Membranes

CMs (GENOSS, Suwon, Korea) and EI-BCMs (Jadam Co., Jeju, Korea) were chosen as barrier membranes. The bacterial strain *Gluconacetobacter hansenii TL-2C* was incubated for 7 days in a static culture containing 0.3% (*w*/*w*) citrus fermented solution and 5% (*w*/*w* sucrose of pH 4.5 (adjusted using acetic acid). The obtained gel-like pellicles of BC were purified by immersion in deionized water at 90 °C for 2 h and boiling in 0.5 M aqueous NaOH for 15 min to remove bacterial cell remnants. The BC obtained was washed with deionized water several times and soaked in 1% NaOH for 2 days.

### 4.2. Fabrication of Electron Beam Irradiated Bacterial Cellulose Membranes

**Figure 10:**
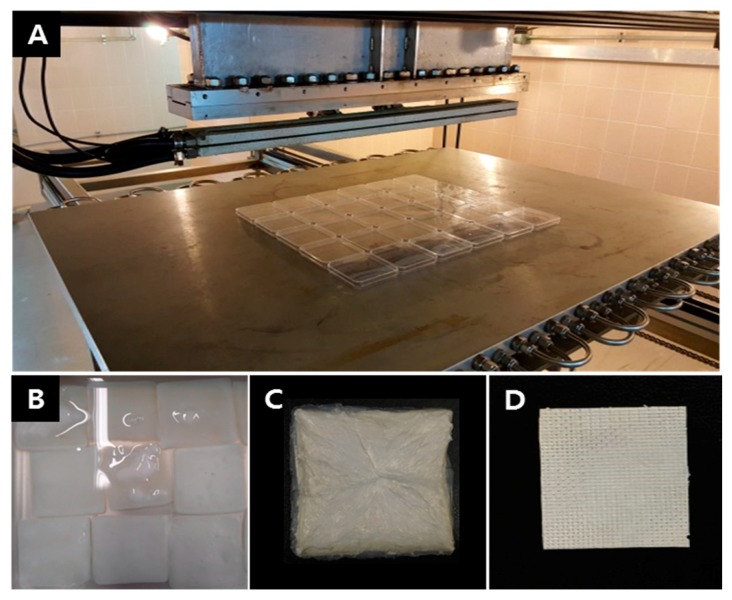
(**A**) Electron beam linear accelerator (Korean Atomic Energy Research Institute, Jeongeup, Korea); (**B**) BC pellicles were irradiated to 100 kGy in distilled water; (**C**) The electron beam irradiation BC pellicles after freeze-drying; (**D**) The electron beam irradiated BC membrane obtained by compression.

**Figure 11:**
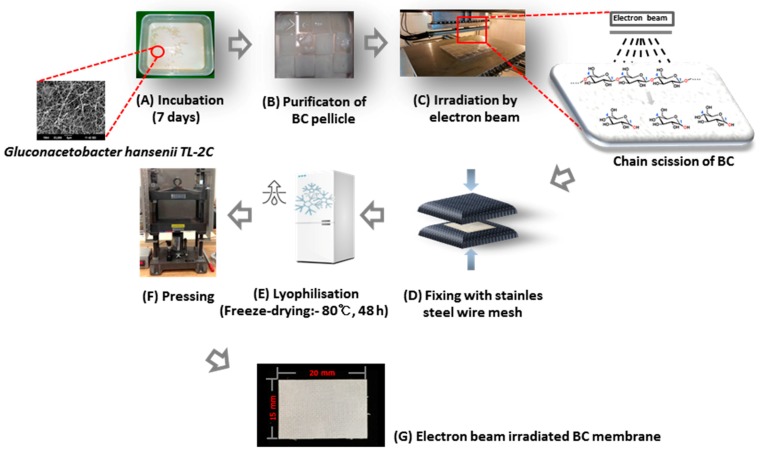
Fabrication of electron beam irradiated BC membranes.

BC pellicles were first washed with distilled water, irradiated at 5 kGy/min to 100 kGy at room temperature using an electron beam linear accelerator (10 MeV, 0.5 mA) at the Korean Atomic Energy Research Institute, washed with deionized water, fixed between stainless steel wire meshes to remove water, dried in a freeze dryer at −80 °C for 48 h, and compressed into sheet using pressing machine (Carver 3969, Wabash, IN, USA) at room temperature for 5 min to produce EI-BCMs. All other reagents and solvents were of analytical grade and used without further purification (Figure 10 and Figure 11).

### 4.3. In Vitro Mechanical Studies

#### 4.3.1. Scanning Electron Microscope (SEM) Analysis

SEM images of CMs and EI-BCMs were obtained using a JSM-6390 unit (JEOL, Tokyo, Japan) at 10 kV and a distance of 10–12 mm. Samples were deposited on a steel plate and sputter coated with gold for 60 s.

#### 4.3.2. Mechanical Strength Measurements

The mechanical properties of CMs and EI-BCMs were evaluated by a Universal Testing machine (Instron 5569, Instron Corp., Canton, OH, USA) using a 5 kN load cell and crosshead speed of 10 mm/min. The samples were cut into 20 mm × 15 mm pieces. This method specifies a procedure for determining the wet tensile strength through measuring the tensile strength of the samples according to ASTM standard method D 882-88 after being soaked in water for 10 min.

#### 4.3.3. Porosity Analysis

The porosities and pore-size distributions of CMs and EI-BCMs were determined using a mercury porosimeter (AutoPore IV 9500, Micromeritics Instrument Corp., Norcross, GA, USA). The maximum application pressure of mercury was 31,000 psi (214 MPa). Mercury-intrusion measurements were corrected for the compression of liquid mercury and the expansion of the penetrometer (sample holder). Detailed working information of the mercury porosimeter used can be obtained from the manufacturer.

### 4.4. In Vitro Cell Studies

#### 4.4.1. Cell Culture

NIH3T3 cells (ATCC^®^ CRL-1658™, mouse embryo fibroblasts) were cultured in Dulbecco’s Modified Eagle Medium containing 4.5 g·L^−1^ glucose (DMEM-HG, Gibco BRL, Grand Island, NY, USA) and supplemented with 10% fetal bovine serum and 1% penicillin/streptomycin in a 5% CO_2_ incubator at 37 °C and 95% RH. Medium was changed every two days.

#### 4.4.2. Cell Proliferation Assay

Cell proliferation was measured using a Cell Counting Kit-8 assay (CCK-8, Dojindo Laboratories, Kumamoto, Japan). NIH3T3 cells were seeded at a density of 1 × 10^5^ cells/well on CM or EI-BCM surfaces, and then cultured for 1, 3, or 7 days. Culturing media were exchanged with culture medium containing 10% CCK-8 solution. Then, after maintaining conditions for 90 min, absorbance was measured at 450 nm using a UV-Vis spectrophotometer (MQX 200, Bio-Tek Instruments, Winooski, VT, USA). All experiments were performed in triplicate.

#### 4.4.3. Immunofluorescent Staining

Cell nuclei and F-actin were stained to evaluate the morphologies of cells on membranes. After culturing for 24 h, samples were fixed using 3.7% MeOH-free formaldehyde in PBS for 10 min at 37 °C, wash with PBS, permeabilized in cytoskeleton (CSK) buffer (10.3 g sucrose, 0.292 g NaCl, 0.06 g MgCl_2_, 0.476 g HEPES buffer, 0.5 mL Triton X-100, in 100 mL water, pH 7.2) for 10 min at 4 °C, and blocked in blocking buffer (1% BSA in PBS) for 1 h at 37 °C. Samples were then incubated with Rhodamine-phalloidin (1:100) and Hoechst 33258 (1:1000) (nuclear stains; both from Molecular Probes, Eugene, OR, USA), for 1 h at 37 °C. After washing in PBS, samples were mounted on glass slides. Fluorescent images of stained cells on membranes were acquired using a Laser Scanning Confocal Microscope (LSM 510, Zeiss, Jena, Germany). Projected cell areas in acquired images were determined using Image proPlus 4.5 (Media Cybernetics, Silver Springs, MD, USA).

### 4.5. In Vivo Animal Studies

#### 4.5.1. Experimental Animals

Two systemically healthy male beagle dogs, 18 months old and approximately 10 kg in weight, were chosen for this study. The animals were fed a soft food diet to preserve dentition and healthy periodontium. Animal selection and management and surgical procedures were conducted with the approval of the Ethics Committee on Animal Experimentation at Chonnam National University (CNU IACUCTB-2013-10). All experiments were performed at the animal dental laboratory accredited by Chonnam National University Animal Hospital.

#### 4.5.2. Surgical Procedures for Tooth Extraction

Second and fourth premolars (P2 and P4) of mandibles were extracted under general anesthesia during first surgery; animals were fasted for 12 h before general anesthesia. Cimetidine (H-2 Amp.; 5 mg·kg^−1^, IV, JW Pharmaceutical, Korea), cefazolin (Cefozol Inj.; 20 mg·kg^−1^, IV, Hankook Korus Pharm, Seoul, Korea) and enrofloxacin (Baytril Inj.; 5 mg·kg^−1^, SC, Bayer, Seoul, Korea) were used for pre-anesthetic medication. Induction of anesthesia was performed by injecting medetomidine (Domitor Inj.; 48 µg·kg^−1^, Pfizer Animal Health Korea, Seoul, Korea), tiletamin/zolazepam (Zoletil Inj.; 3 mg·kg^−1^, Virbac Korea, Seoul, Korea) and tramadol hydrochloride (Maritrol Inj.; 5.4 mg·kg^−1^, Jeil Pharmaceutical, Daegu, Korea) in the same syringe intramuscularly. After intubation, general anesthesia was maintained by sevoflurane (Sojourn; 2–3.5%, Piramal, Bethlehem, PA, USA) and 0.9% normal saline (Daihan Sterile Normal ay Saline Inj.; 10 mL·kg^−1^·h^−1^, IV, Daihan Pharm, Seoul, Korea) during surgery. Ketamine CRI (Yuhan Ketamine Inj.; 12 mg·kg^−1^·min^−1^, Yuhan, Seoul, Korea) and tramadol (Maritrol Inj.; 2 mg·kg^−1^, Jeil Pharmaceutical, Daegu, Korea) were administered i.v. to control pain. At surgical sites, 1 mL 2% lidocaine HCl and 1:100,000 epinephrine (Yu-Han Co., Gunpo, Korea) was used for dental infiltration anesthesia. Targeted premolars were extracted bilaterally, and extraction sites were sutured with 4-0 nylon (Mersilk, Ethicon Co., Livingston, UK). Oral prophylaxis was performed on remaining teeth. Stitches were removed after 10 days and extraction sites were allowed to heal.

#### 4.5.3. Surgical Procedures for Implant Placement and Guided Bone Regeneration (GBR)

**Figure 12:**
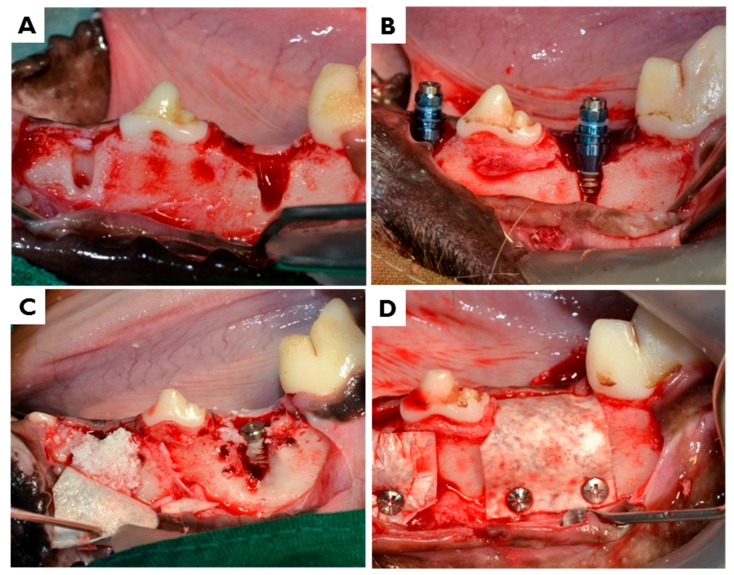
Surgical procedures: (**A**) The mucoperiosteal flaps were elevated to expose the edentulous alveolar ridge and buccal dehiscence defects (4 mm high and 2 mm wide) were created; (**B**) implants (3.0 mm in diameter and 6.0 mm in length) were inserted; (**C**) 0.1 mg deproteinized bovine bone grafting material (Bio-Oss) was positioned at each buccal dehiscence defect; and (**D**) CMs or EI-BCMs were randomly applied at buccal dehiscence defects filled with grafting materials.

Second surgeries proceeded after an 8-week healing period. General anesthesia and local infiltration anesthesia were performed as in first surgeries. In each animal, a horizontal incision was made on the experimental site and vertical incisions were made through the mucogingival junction that extended into the alveolar mucosa at the mesial and distal ends of each defect. Mucoperiosteal flaps were elevated to expose the edentulous alveolar ridge, and two identical dehiscence defects (4 mm in height and 2 mm in width) were surgically prepared on the buccal sides of the left and right partial edentulous ridges. Four peri-implant dehiscence defects were prepared per animal. Implants were placed by serial drilling using to a standard protocol. A total of 8 implants (3.0 mm in diameter and 6.0 mm in length; INNO Implant, Cowellmedi Co., Busan, Korea) were bilaterally placed in dehiscence defects in each animal. Fixture platforms were located at the level of the alveolar crest. Deproteinized bovine bone grafting material (0.1 mg; Bio-Oss, Geistlich Biomaterials, Wolhusen, Switzerland) was weighed out, dampened with sterile saline for 5 min, and then positioned at each buccal dehiscence defect. Upon completing grafting, CMs (CM; GENOSS, Suwon, Korea) or electron beam irradiated BC membranes (EI-BCM; Jadam Co., Jeju, Korea) were randomly applied at buccal defects. All membranes were cut to cover 2–3 mm of adjacent alveolar bone to overlap entire defects (Figure 12). To enhance graft material stability, titanium pins (Dentium Co., Seoul, Korea) were used for the fixation. To obtain primary wound closure, buccal flaps were carefully released and sutured with 4-0 nylon (Mersilk, Ethicon Co., Livingston, UK). All surgeries were performed by the same professionally trained operator.

#### 4.5.4. Post-Operative Care and Sacrifice

After surgery, animals were fed with amoxicillin-clavulate acid (Lactamox Tab.; 12.5 mg·kg^−1^, BID, PO, Boryung, Korea), firocoxib (Previcox Tab.; 2.5 mg·kg^−1^, BID, PO, Merial, Lyon, France), famotidine (Famotidine Tab.; 0.5 1 mg·kg^−1^, BID, PO, Korea Nelson Pharmaceutical, Seoul, Korea) and milk thistle (Silymarin Tab.; 51 mg·kg^−1^, BID, PO, Sinil Pharmaceutical, Seoul, Korea) for two weeks with a soft diet. Oral gel (MAXI/Guard OraZn; 2 times·day^−1^, Addison Biological Laboratory, Fayette, MO, USA) was applied to mouths until sacrifice. All animals were sacrificed at 8 weeks post-operatively by intravenous concentrated sodium pentobarbital injection (Euthasol, Delmarva Laboratories Inc., Midlothian, VA, USA). The mandibles of sacrificed dogs were harvested with alveolar bones near implants and membranes and surrounding mucosae. The eight harvested mandible block sections were fixed in neutral buffered formalin (Sigma Aldrich Co., St. Louis, MO, USA).

#### 4.5.5. Micro-Computed Tomography (μCT) Analysis

**Figure 13:**
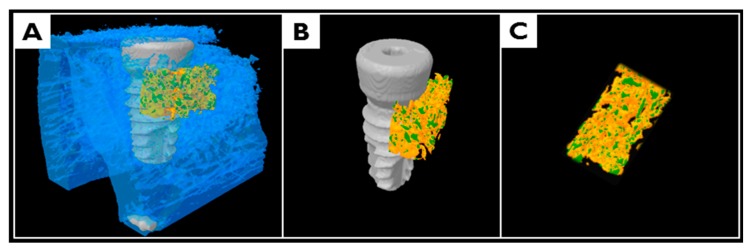
3D images obtained by micro-computed tomography (μCT); (**A**) mandibular block section around a peri-implant; (**B**) new bone in a peri-implant without the old bone; and (**C**) new bone of region of interest (ROI).

**Figure 14:**
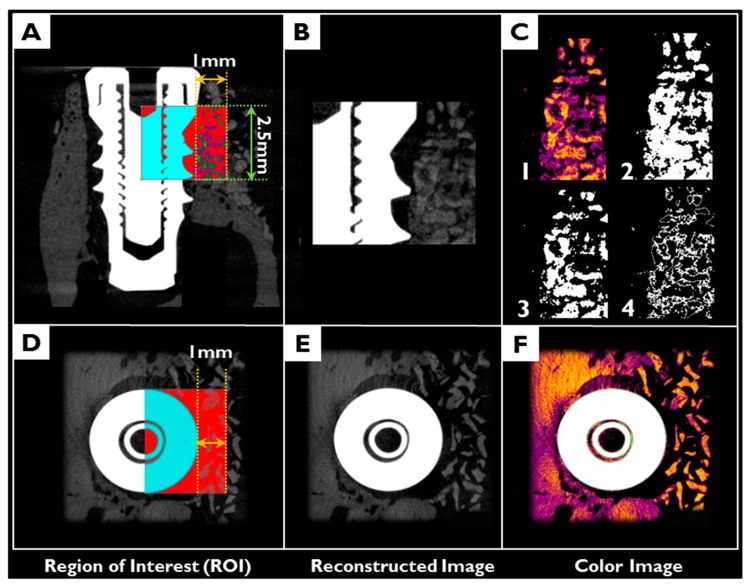
3D reconstructed μCT images of a region of interest (ROI: 1 mm wide and 2.5 mm high from the implant platform): (**A**–**C**) cross sectional view; (**D**–**F**) occlusal view; (**C1**,**2**) images of total bone including bone graft materials (**C3**); and new bone (**C4**).

After fixation, 3D μCT images were generated to analyze new bone densities and new bone volumes in peri-implant dehiscence defect areas (Figure 13). Specimens were wrapped with Parafilm M^®^ (Bemis Company Inc., Neenah, WI, USA) to keep them from drying during the scanning process. Samples were scanned at 130 kV, 60 μA, at a pixel resolution of 57.10 μm using a bromine filter (0.25 mm) of high energy μCT (Skyscan 1173, version 1.6, Bruker-CT, Kontich, Belgium). Reconstruction was performed using Nrecon software (version 1.6.10.1, Bruker-CT, Kontich, Belgium). Applied scan and reconstruction parameters were identical for all specimens. 3D reconstructed view was used to evaluate augmented bone contours. Boundaries were set to standardize the region of the augmented volume for analysis. Regions of interest (ROIs) were 1 mm wide and 2.5 mm high from implant platforms (Figure 14).

The following parameters were calculated within ROIs

- Total augmented volume (TAV; mm^3^): volume occupied by total augmented bone volume within the ROI
- Non-mineralized tissue volume (NMV; mm^3^): volume occupied by non-mineralized tissue volume within the ROI
- Remaining bone substitute volume (RBV; mm^3^): volume occupied by remaining bone substitute volume within the ROI
- New bone volume (NBV; mm^3^): volume occupied by the new bone volume within the ROI

#### 4.5.6. Histomorphometric Analysis

**Figure 15:**
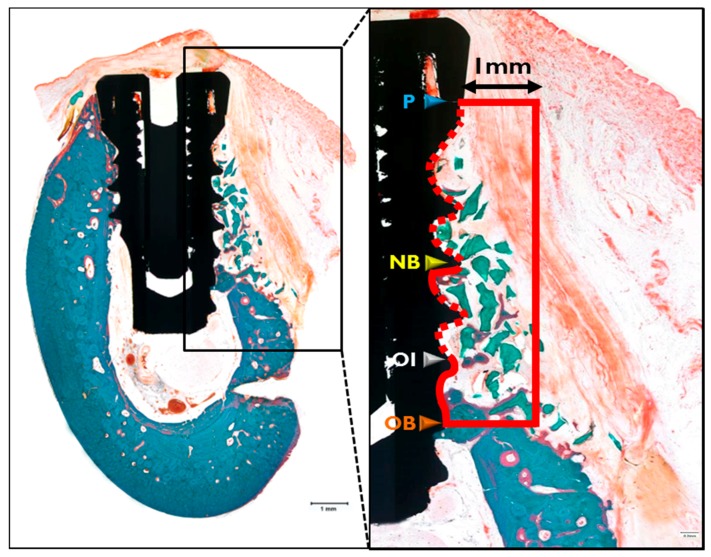
Parameters measured in histologic specimens. Red box, the area of interest (AOI was 1 mm wide and 1 mm high from the implant platform to the uppermost point of old bone (P-OB)); Blue arrow, platform of the implant (P); Yellow arrow, the uppermost point of new bone (NB); White arrow, uppermost point of the osseointegration site (OI); Orange arrow, uppermost point of old bone (OB) in the AOI.

After μCT analysis, specimens were cleansed and dehydrated using an ethanol series, and then infiltrated with an ethanol/Technovit 7200 resin (Heraeus Kulzer, Hanau, Germany) ladder at increasing resin ratios. Specimens were then fixed on an embedding frame and embedded using a UV embedding system (Exakt 520, Kulzer) according to the manufacturer’s instructions. Polymerized specimen blocks were longitudinally sectioned at each implant center at 400 μm using the Exakt diamond cutting system (Kulzer Exakt 300 CP), and sections were attached to slides using an adhesive press system. Final slides were ground to a section thickness of 40 ± 5 μm using the Exakt grinding system (Kulzer Exakt 400CS). To observe newly regenerated bone in specimens, Goldner Trichrome staining was conducted before mounting sections. Images were captured using a CCD camera (Spot Insight 2 Mp, Diagnostic Instruments Inc., Sterling Heights, MI, USA) equipped with an adaptor (U-CMA3, Olympus, Tokyo, Japan) mounted on a light microscope coupled to a computer (BX51, Olympus). To analyze captured images, i-Solution ver. 8.1 (IMT i-Solution Inc., Coquitlam, BC, Canada) was used. Specimens were observed at 12.5× and histometric analysis was performed at 40×. A professionally trained investigator measured the following items. Areas of interest (AOI) were 1 mm wide and 1 mm high from the implant platform (P) to the most point of the old bone (OB) (Figure 15).

The following measurements were made and recorded.

- New bone area (NBA; %): Area occupied by the new bone/AOI × 100
- Remaining bone substitute area (RBA; %): Area occupied by the remaining bone substitute/AOI × 100
- Bone-to-implant contact (BIC; %): Length of contact with new bone/total length of exposed threads × 100
- New bone-old bone (NB-OB; %): Distance from the most upper point of new bone to the most upper point of old bone /P-OB × 100
- New bone-old bone (NB-OB; %): Distance from the most upper point of new bone to the most upper point of old bone /P-OB × 100
- Osseointegration-old bone (OI-OB; %): Distance from the most upper point of osseointegration to the most upper point of old bone /P-OB × 100

### 4.6. Statistical Analysis

All results were obtained from sample analysis performed in triplicate. Results are presented as means ± SDs. Because of deviations from normal distribution, non-parametric tests were used. The analysis was performed using SPSS ver. 21.0 (SPSS, Chicago, IL, USA). The Mann–Whitney U test with post hoc analysis was used to determine the significances of differences. Statistical significance was accepted for *p* values < 0.05.

## 5. Conclusions

The lack of biodegradability of bacterial cellulose restricts its usefulness as a resorbable barrier membrane for bone tissue regeneration. To overcome this limitation, we exposed BCMs to an electron beam. Mechanical strength, cell adhesion and proliferation, and bone regeneration in peri-implant bone defects showed irradiated EI-BCMs have the potential to replace existing resorbable barrier membranes.

## Figures and Tables

**Table 1:** Volumetric analysis within areas of interest (AOIs) (*n* = 4).

Group (Membrane)	NBV (mm^3^)	TAV (mm^3^)	RBV (mm^3^)	NMV (mm^3^)
CM	1.23 ± 0.86	14.28 ± 0.17	2.40 ± 2.36	10.65 ± 3.23
EI-BCM	1.14 ± 0.91	14.41 ± 0.16	2.31 ± 2.04	10.96 ± 2.85
*p*	>0.05	>0.05	>0.05	>0.05

CM, collagen membrane; EI-BCM, electron beam irradiated bacterial cellulose membrane; NBV, new bone volume; TAV, total augmented volume; RBV, remaining bone substitute volume; NMV, non-mineralized tissue volume. No significant difference between the two groups (*p* > 0.05).

**Table 2:** Histometric analysis within areas of interest (*n* = 4).

Group (Membrane)	NBA (%)	RBA (%)	BIC (%)	NB-OB (%)	OI-OB (%)
CM	15.07 ± 9.95	12.49 ± 7.57	41.33 ± 13.52	54.67 ± 31.78	37.40 ± 16.63
EI-BCM	16.51 ± 13.00	9.05 ± 11.61	38.82 ± 25.74	44.35 ± 39.90	38.52 ± 37.60
*p*	>0.05	>0.05	>0.05	>0.05	>0.05

CM, collagen membrane; EI-BCM, electron beam irradiated bacterial cellulose membrane; NBA, new bone area; RBA, remaining bone substitute area; BIC, bone-to-implant contact; NB-OB, distance from the most upper point of new bone to old bone; OI-OB, distance from the most upper point of the osseointegration to old bone. No significant difference between CM and EI-BCM transplanted animals (*p* > 0.05).
